# Site-selective substitutional doping with atomic precision on stepped Al (111) surface by single-atom manipulation

**DOI:** 10.1186/1556-276X-9-235

**Published:** 2014-05-13

**Authors:** Chang Chen, Jinhu Zhang, Guofeng Dong, Hezhu Shao, Bo-yuan Ning, Li Zhao, Xi-jing Ning, Jun Zhuang

**Affiliations:** 1 Shanghai Engineering Research Center of Ultra-Precision Optical Manufacturing, Department of Optical Science and Engineering, Fudan University, Shanghai 200433, China; 2 State Key Laboratory of Surface Physics, Department of Physics, Fudan University, Shanghai 200433, China; 3 Applied Ion Beam Physics Laboratory, Institute of Modern Physics, Department of Nuclear Science and Technology, Fudan University, Shanghai 200433, China

**Keywords:** Single-atom doping, Substitutional, Single-atom manipulation, Atomic precision, Metal surface

## Abstract

In fabrication of nano- and quantum devices, it is sometimes critical to position individual dopants at certain sites precisely to obtain the specific or enhanced functionalities. With first-principles simulations, we propose a method for substitutional doping of individual atom at a certain position on a stepped metal surface by single-atom manipulation. A selected atom at the step of Al (111) surface could be extracted vertically with an Al trimer-apex tip, and then the dopant atom will be positioned to this site. The details of the entire process including potential energy curves are given, which suggests the reliability of the proposed single-atom doping method.

## Background

Single-atom manipulation, which was first introduced by Eigler et al*.* and realized experimentally on Ni (111) surface with a scanning tunneling microscope (STM) tip, provides a way to fabricate nanostructures with atomic precision [1-7]. Besides the STM tip, for nonconductive surface, the tip of an atomic force microscope (AFM) has also been applied to achieve various single-atom manipulations [8-10]. Studies show that merely by the mechanical interaction force acting between the tip and atom, complex manipulations can still be accomplished besides the primary lateral and vertical manipulations. For instance, on Al (111) surface, a reversible modification of the configuration of supported nanoclusters with atomic precision by tip was demonstrated in our previous simulations [11]. Also, the work on Si (111) surface given by Sugimoto et al*.* shows that an atom from the AFM tip can interchange with a surface adatom in a reversible exchange procedure [9]. Through this vertical manipulation, a single Si atom can be precisely positioned into or extracted from the Sn layer. As the size of devices shrinks to nanoscale or even to atomic scale, besides configuration of nanostructure, the number of isolated atoms of certain species and their location could modify their functionality and performance [12,13]. Therefore, it is sometimes demanded to position dopants at certain sites precisely. For example, by STM-based hydrogen lithography, a single-atom transistor in which an individual P dopant atom has been placed within a silicon device with a spatial accuracy of one lattice site was demonstrated recently [14]. In another work, by Cs atom doping with a STM tip, spin of individual magnetic molecules as basis of quantum computer was successfully controlled [15].

On metal surfaces, influences of tip structure on the manipulation were intensively investigated in our previous work [16], and it was shown that the trimer-apex tip, a model of blunt tip in the experiment, is capable of transforming the configuration of the Al nanocluster reversibly [11]. The specific manipulation procedure also shows that the trimer-apex tip combined with the single-apex tip has potential to achieve single-atom substitutional doping in the edge of the cluster and to change its composition, which is the motivation of the present work. Usually, the edge of the Al nanocluster is modeled by stepped Al (111) surface. The extraction and position processes are studied, wherein the mechanism is the mechanical interaction force acting between the tip apex and surface. An individual atom at the step is extracted first by the tip, and then single Ag or Au dopant is positioned to this site. Based on the first-principles simulation, details of the doping process are given and its reliability is discussed.

## Methods

**Figure 1:**
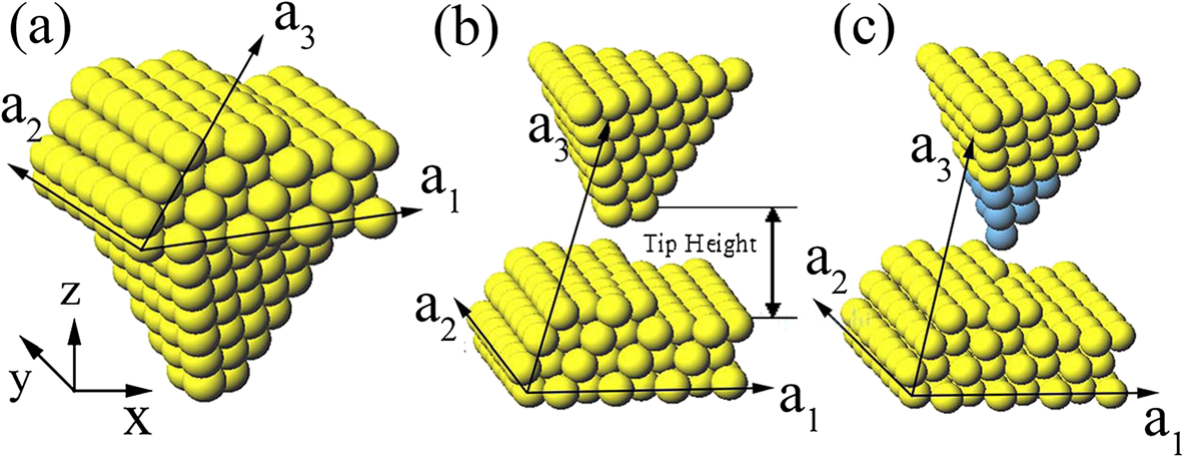
**Simulation model.** The simulation model **(a)** before and **(b)** after the periodic boundary condition is applied, in which the tip is initially placed above the manipulated atom. **(c)** The doping process, where the yellow balls represent Al atoms and blue balls represent dopants such as Ag or Au.

As shown in Figure 1a, the system we studied is modeled by a three-layer Al (111) slab with a step on the top, each layer contains 8 **×** 7 atoms. The pyramidical Al tip is mimicked by six- or seven-layer atoms mounted on the reverse of the slab. In our simulation, two types of tips are considered, single-apex tip and trimer-apex one, which are the models of sharp and blunt tips in the experiment, respectively. Besides Al atoms, different tip apex atoms such as Ag and Au are taken for doping process. In our survey, the tip with different apex atoms can be obtained in the experiment [17,18]. As there are six/seven extra layers for the tip, our model is convergent with the energy error of around 3%. Periodic boundary conditions are imposed both parallel and perpendicular to the surface with the periodic vectors $\vec{a1}$, $\vec{a2}$, and $\vec{a3}$ (see Figure 1a). By applying the periodic boundary condition in *Z* direction and adjusting $\vec{a3}$, as shown in Figure 1b, we can move the tip above the stepped surface. The tip is initially placed above the step row at a certain height. The distance between the tip apex atom and the surface of the lower terrace, which is defined as the tip height, is deduced from the *Z* component of the periodic vector $\vec{a3}$ (see Figure 1b).

In manipulations, the tip is moved along the *X* or *Z* direction in a certain step by changing the corresponding components of $\vec{a3}$ accordingly. Manipulations are simulated by molecular statics method: after each step, all atoms except the ones in the bottom layer of the slab and the top layer of the tip are fully relaxed until the forces are smaller than 0.01 eV/Å. Simulations are based on density functional theory (DFT) employing the Vienna *ab initio* simulation program (VASP) [19]. The exchange-correlation potential is described by the generalized gradient approximation [20]. Ultrasoft pseudopotentials are used for the electron-ion interactions with a cutoff energy of 129 eV [21]. The Brillouin zone is sampled with 2 **×** 4 **×** 1 *k* points of a Monkhorst-Pack grid. With these parameters, the obtained lattice parameter of Ag is 4.049 Å, which compares well with the experimental value of 4.05 Å.

## Results

**Figure 2:**
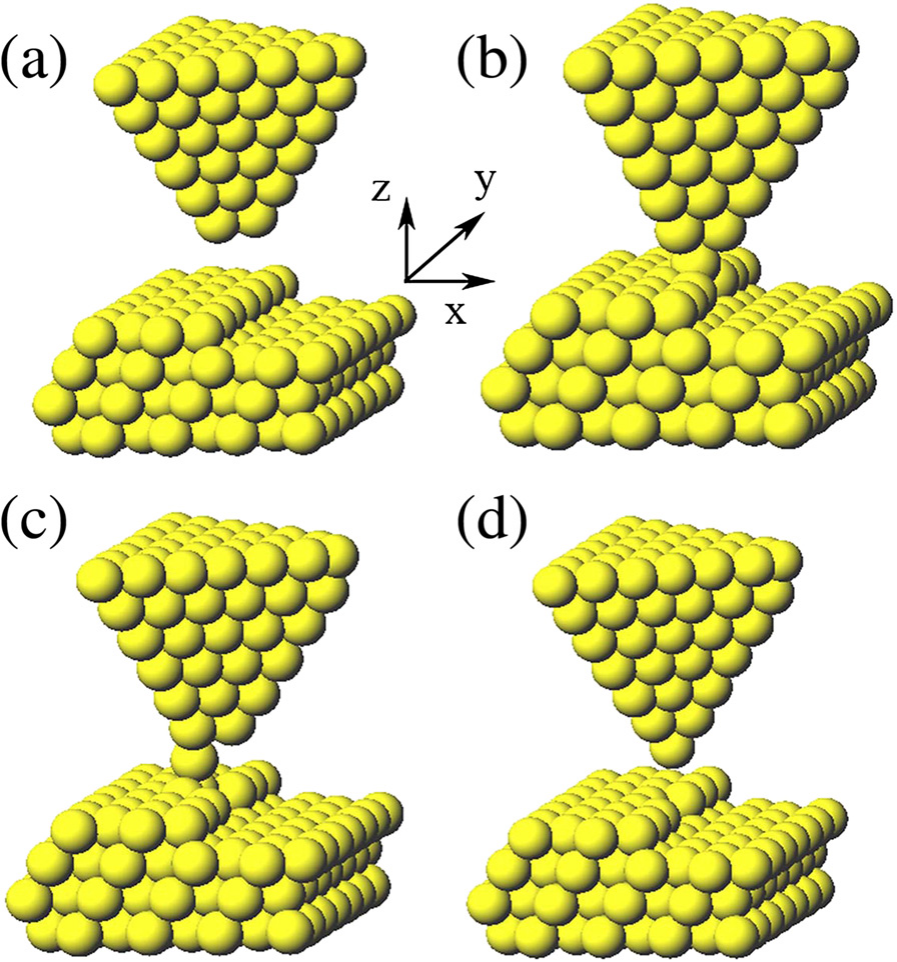
**The process of extracting Al atom from the step row by the trimer-apex tip. (a)** The tip is located upon the manipulated atom. **(b)** Lower down the tip and the manipulated atom rises. **(c)** Lift up the tip gradually. **(d)** Finally, the atom is completely extracted from the step site and adsorbed on the tip.

For the substitutional doping, the first step is extraction of surface atom. For this purpose, we consider the trimer-apex tip due to its strong attraction to the surface atom [11]. Initially, the tip is placed above the manipulated atom high enough so that the tip-surface interaction is almost negligible, as shown in Figure 2a. Then, we lower down the tip step by step. The manipulated atom in the step row rises slightly as the tip approaches the surface. When the tip height reaches 5.9 Å, as shown in Figure 2b, the atom is pulled up obviously from the initial site. After that, we lift up the tip gradually as shown in Figure 2c to Figure 2d; finally, the atom is completely extracted from the step site and adsorbed on the tip. During the whole process, the tip experiences almost no distortion, which indicates that it is stable enough against the atomic interactions with the surface. In addition, in the extracting process, the neighbor atoms of the manipulated atom do not show any obvious upward motion, which means that the trimer-apex tip can exert effectively attractive force on a single atom to make a precise single-atom extraction.

**Figure 3:**
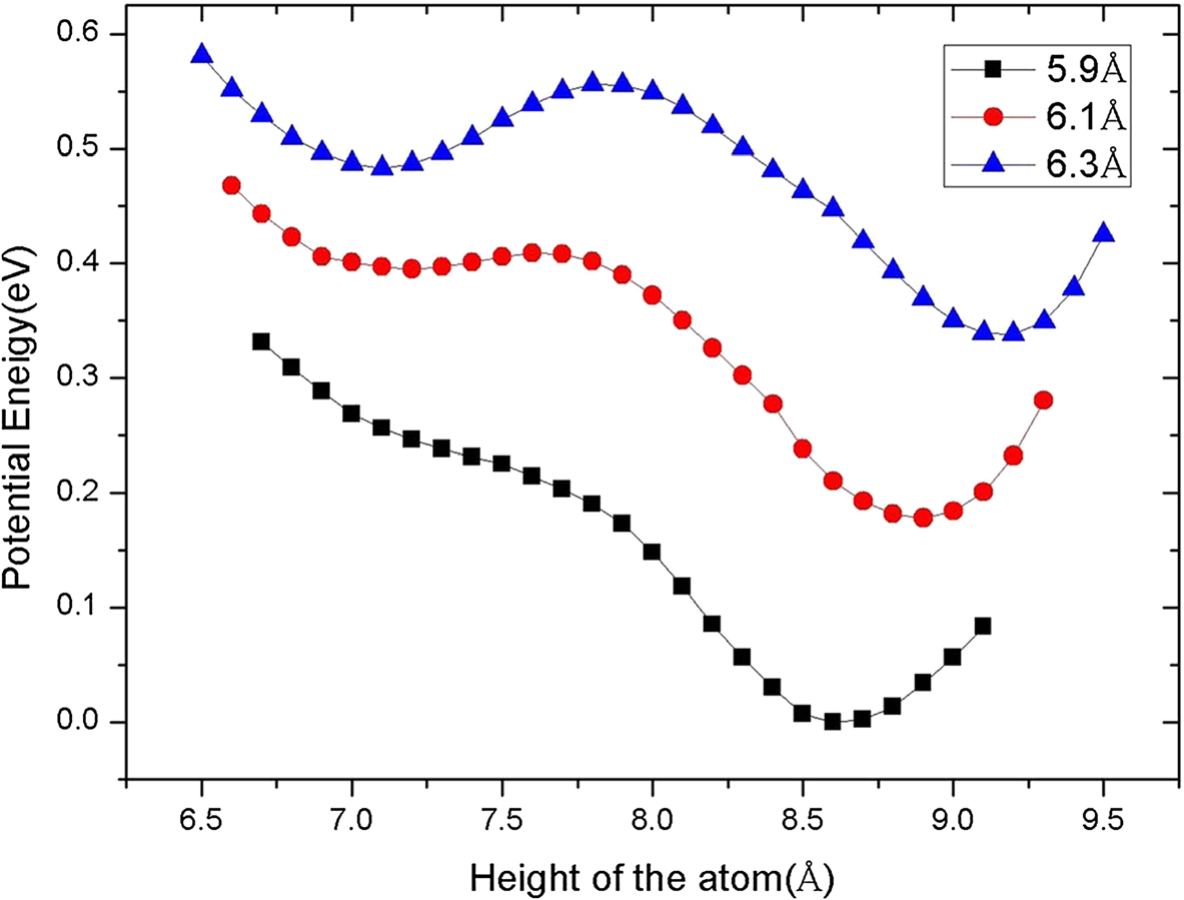
**Variation of potential energy relative to height of manipulated atom.** At different tip heights, the relative potential energy varies with the height of the manipulated atom from the Al (111) step surface.

For understanding the extraction process, as shown in Figure 3, we give the total energy varying with the height of the manipulated atom relative to the bottom of the slab when the tip height is fixed at different heights. That is, at a certain tip height, we move the manipulated atom down from above in a step of 0.1 Å, and at every step, the system is relaxed thoroughly. The figure shows that at the tip height greater than about 6.3 Å, there are two local minimum energy wells: one near the surface and the other near the tip. When the tip height is lower than 6.3 Å, the well near the surface disappears gradually. At 5.9 Å, as shown in Figure 3, there is only one well near the tip, which means that the manipulated atom originally in the step will jump to the well near the tip. This is the reason why the manipulated atom obviously moves toward the tip when the tip reaches the height of 5.9 Å. After that, if we lift up the tip, the curves in Figure 3 indicate that the manipulated atom will stay in the well near the tip. That is, the atom will follow the tip and be extracted from the surface, as the simulation above shows. From Figure 3, we can also estimate the reliability of the extraction process; the energy curve of 6.1 Å shows that the energy barrier for the manipulated atom escaping from the tip is about 0.25 eV, which indicates that the picking up process is robust against the disturbances such as thermal diffusion of atoms.

**Figure 4:**
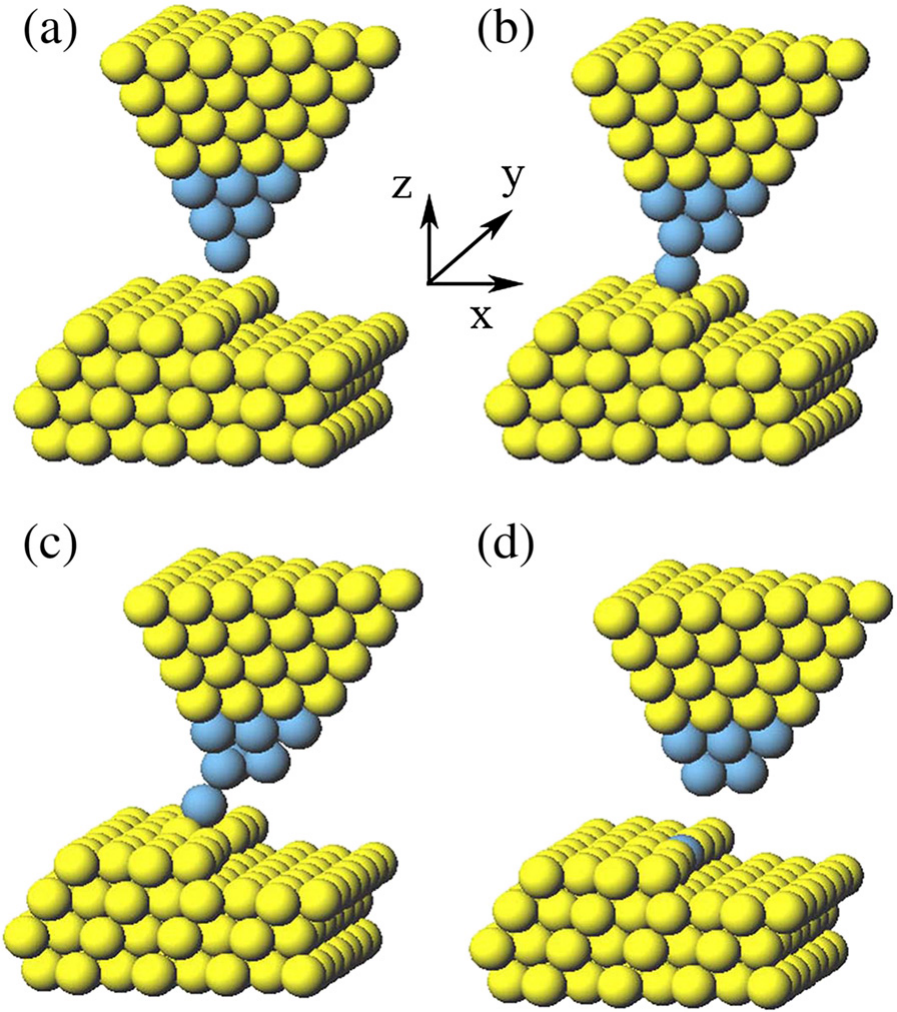
**The process of positioning Ag dopant to the step site by Ag single-apex tip. (a)** The tip is located upon the site. **(b)** As the tip approaches the surface, the dopant atom relaxes toward the up terrace. **(c)** Move the tip laterally in the *X* direction. **(d)** In the end, the dopant atom is released successfully from the tip and adsorbed at the step site.

The next step of substitutional doping is to position a dopant atom to the vacancy site where the Al atom is extracted. Here, we consider two kinds of dopants: Ag and Au atoms. For this purpose, sharp Ag and Au tips with single apex atom are considered; such sharp tip can be fabricated by electroplating and then annealing, or touching a certain metal surface [17,18]. In our simulation, the sharp Ag tip is modeled by a heterogeneous one which contains both Ag and Al atoms, as shown in Figure 4. Blue balls indicate the Ag atoms. The apex of heterogeneous tip is mimicked by three layers of Ag atoms, and our test calculations show that three layers of Ag atoms are equivalent to four layers or more. In other words, three layers of Ag atoms are sufficient for simulation of the sharp Ag tip which is also suitable for the Au tip.

As shown in Figure 4a, the tip is initially placed above the vacancy site with the tip height of 8 Å at which the tip-surface interaction is almost negligible. As the tip approaches the surface step by step, the tip apex atom, i.e., the dopant atom, relaxes toward the up terrace due to the strong attraction. When the tip reaches the height of 7.1 Å, as demonstrated in Figure 4b, the dopant atom shows an obvious movement toward the up terrace since the attraction is strong enough. At this moment, two up-terrace atoms are pulled up slightly and in contact with the dopant atom (see Figure 4b). After that, we move the tip laterally in the *X* direction in a step of 0.2 Å at a constant height. As the tip moves forward, as shown in Figure 4c, the dopant atom drops gradually because of the decreasing vertical attraction from the tip. In the end, the dopant atom is released successfully from the tip and adsorbed at the step site (see Figure 4d). So far, the substitutional doping of the single atom is completed.

**Figure 5:**
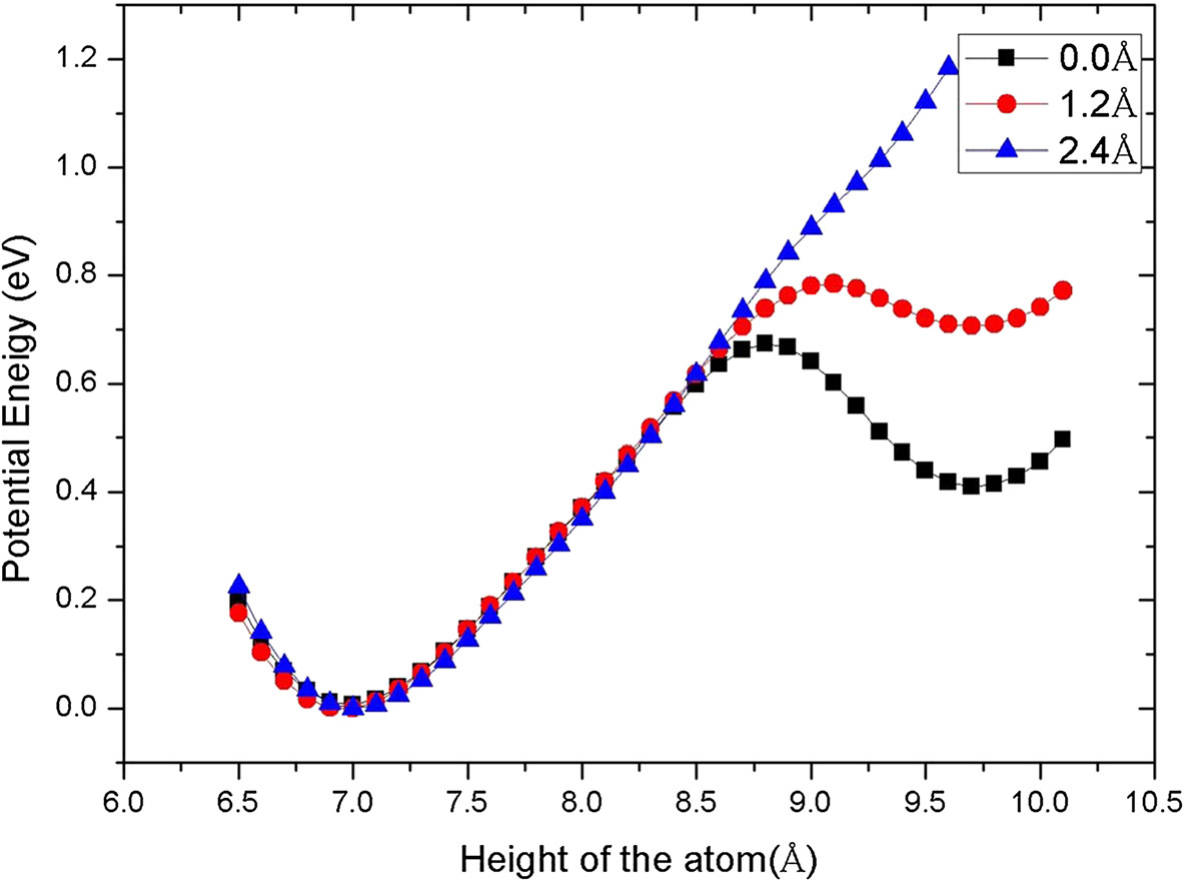
**Variation of potential energy relative to height of dopant atom.** At different lateral distances relative to the vacancy in the *X* direction, the potential energy varies with the height of the dopant atom between the Al (111) surface and the tip.

We also give the total energy curves to understand the doping process in which the tip laterally moves at a constant height of 7.1 Å. In this case, we fix the tip at different lateral distances and move the dopant atom down from above in step of 0.1 Å, and at every step, the system is relaxed thoroughly. The results, presented in Figure 5, show that when the tip stays right upon the vacancy or adsorption site, i.e., the lateral distance is 0.0 Å, there are two local minimum energy wells: one near the surface and the other near the tip like the picking up process. The dopant atom is still located at the tip because of the energy barrier. As the tip moves forward along the *X* direction, the right well disappears gradually which means that the attraction from the tip apex is weakened. At the lateral distance of 2.4 Å, the two wells merge so that the atom jumps to the surface. From the curves in Figure 5, it can be estimated that the energy barrier for the dopant to escape from the step site is greater than 0.6 eV, which indicates that the releasing processes are also reliable even in the elevated temperature.

**Figure 6:**
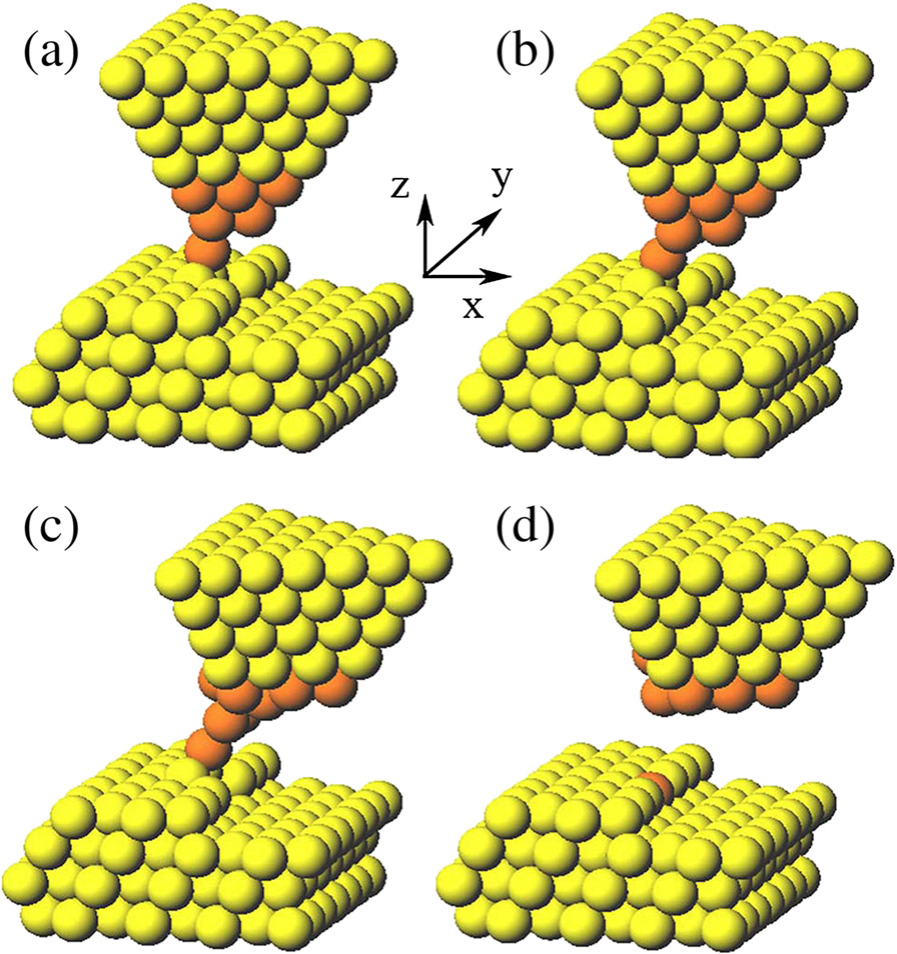
**The process of positioning Au dopant to the Al step site by Au single-apex tip. (a)** Lower down the tip upon the site. **(b)** Move the tip laterally in the *X* direction. **(c)** The Au tip is deformed while moving laterally. **(d)** The dopant atom is released successfully.

In order to check the general applicability of our substitutional doping method, we next consider the Au dopant. Similar to the Ag dopant, as shown in Figure 6, a single Au atom is also successfully doped into the Al stepped surface in the substitutional way. The only difference from the case of the Ag dopant is that the Au tip is deformed after doping.

## Discussion

In our doping, both extraction and reposition processes only rely on the mechanical interaction force acting between the tip apex and the surface. It means that our doping scheme, in principle, can be performed with STM or AFM. For the STM tip, the electric field is inessential. Certainly, the specific parameters need to be further confirmed in the experiments. In addition, we find that the tip orientation has almost no influence on the doping process; as a result, using the tip rotated by 180° around the *Z* axis, we can still achieve the same results. The insensitivity to the tip orientation is beneficial to the practical experiment.

**Figure 7:**
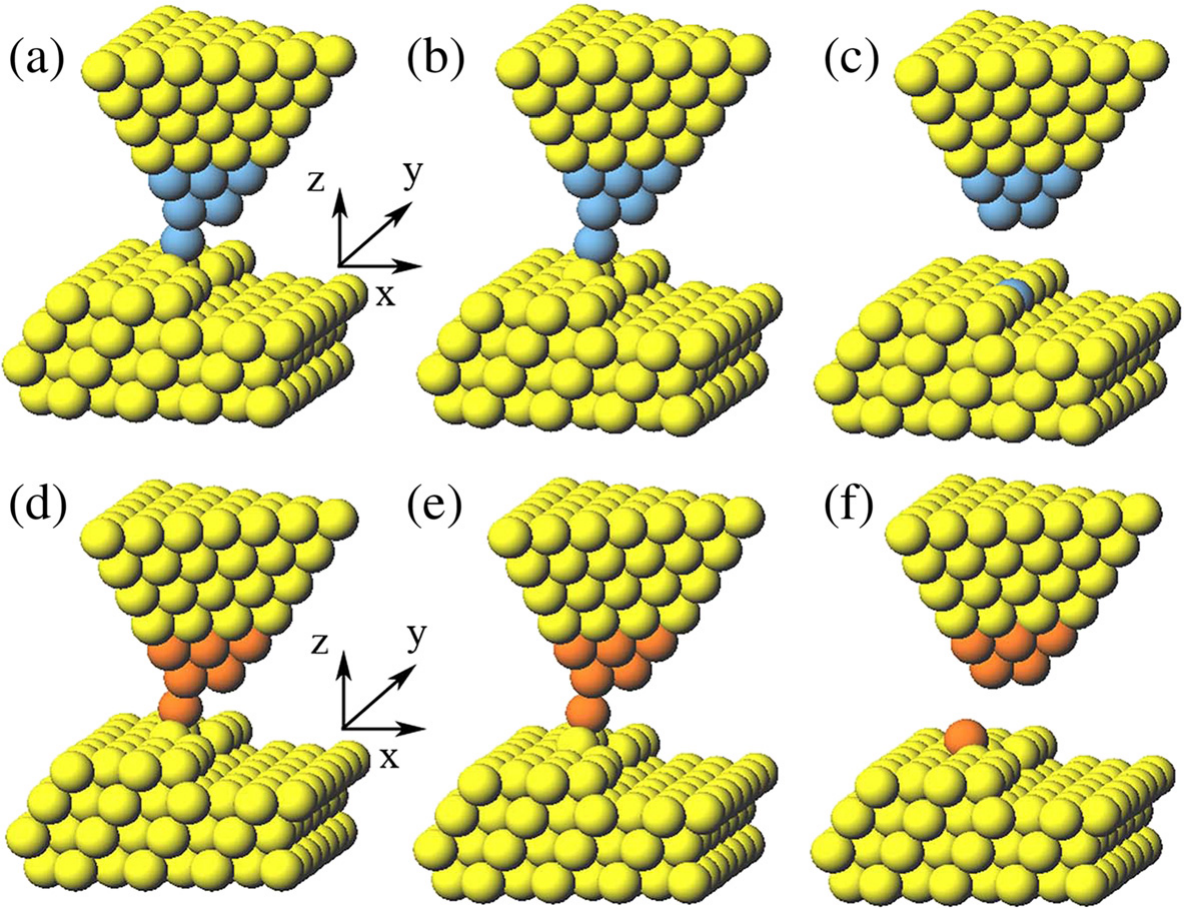
**Withdrawing the tip vertically in *Z *direction to position the dopant. (a-c)** The positioning process of the Ag atom. **(d-f)** The undesirable release of the Au atom.

**Figure 8:**
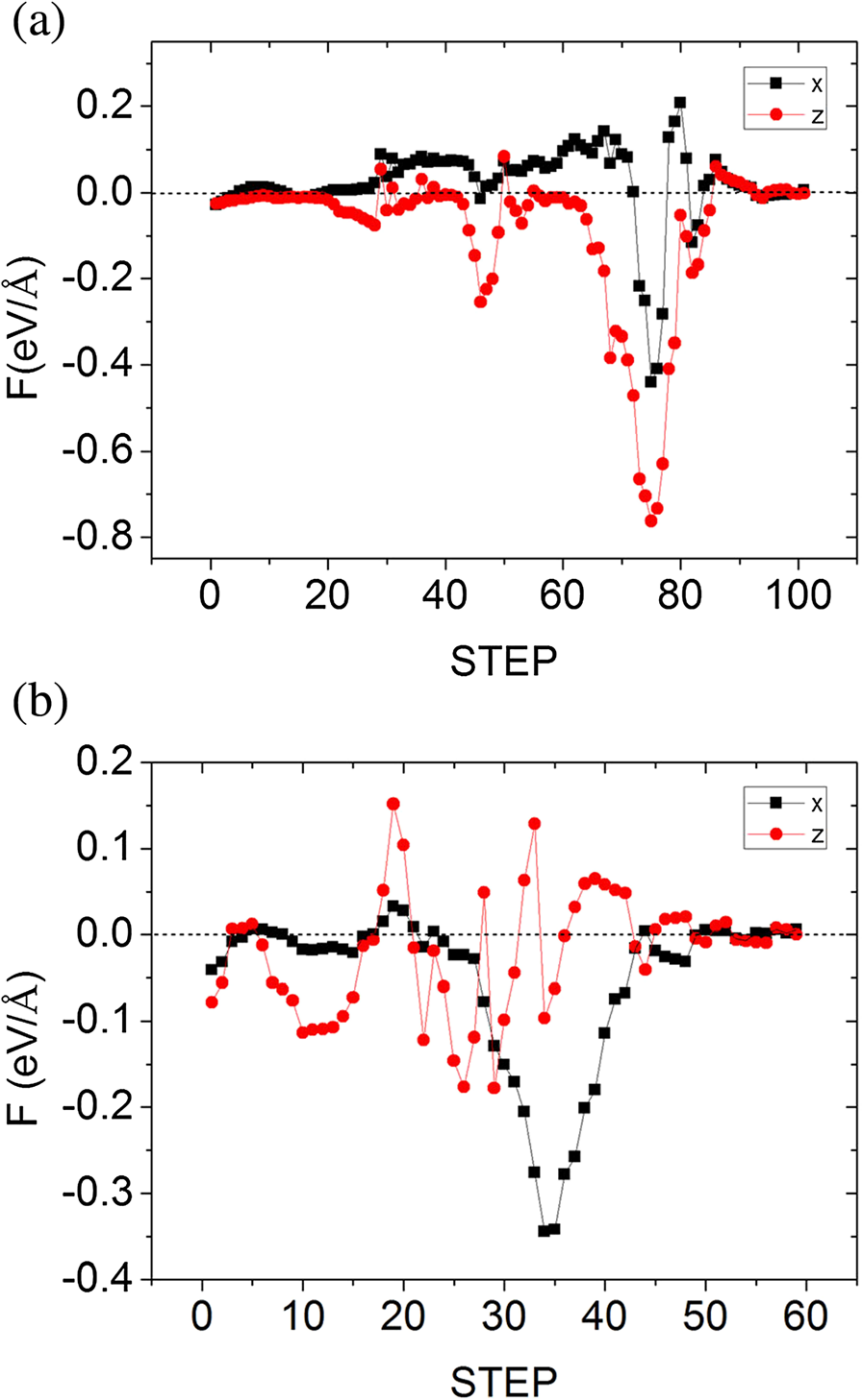
The forces acting on Ag (a) and Au (b) dopant atom in every calculation step **The forces acting on Ag (a) and Au (b) dopant atom in every calculation step.** The red curve is the component force in the *Z* direction. The black curve denotes the component force in the *X* direction.

We also try other approaches to position the dopant. For instance, when the tip reaches 7.1 Å, we withdraw the tip vertically in the *Z* direction instead of moving the tip laterally in the *X* direction. For the Ag dopant, it is positioned to the vacancy site successfully, as shown in Figure 7a,b,c. However, for the Au dopant, it is finally placed on the step terrace, as shown in Figure 7d,e,f. The difference for Ag and Au can be understood from the forces acting on the dopant atom. At the key relax step where the dopant atom falls to the surface, we decompose the forces acting on Ag and Au atoms into the *X* and *Z* directions at every calculation step. The results are shown in Figure 8. For the Ag dopant, the component forces have negative peak values and the one in the *Z* direction is greater than that in the *X* direction, which means that the vertical attraction is greater than the lateral one when the dopant atom is falling. Finally, the Ag atom falls into the step site (see Figure 7c). For the Au dopant, however, the component force in the *X* direction has a greater peak value than that in the *Z* direction. It means that the Au dopant tends to drop onto the step terrace (see Figure 7f). Though withdrawing the tip vertically in the *Z* direction to position the dopant is effective for the Ag atom, it lacks general applicability. Also, the position details and component forces reveal that it is not reliable even in small thermal disturbance (see Figures 7 and 8).

## Conclusion

Based on first-principles simulation, we theoretically investigate the substitutional single-atom doping on stepped Al (111) surface via atomic manipulation. An effective method is proposed in which a trimer-apex tip is adopted to extract the surface atom and then a single-apex one is used to position the single dopant atom. In the positioning process, the tip moves first in the vertical direction and then in a lateral one. Both Ag and Au dopants are successfully positioned to the specific site in atomic precision, which indicates that the method owns a potential of general application. The corresponding energy curves show that both extraction and doping processes have a high reliability against thermal disturbances. Additionally, the manipulation processes are insensitive to the tip orientation, which is beneficial to the realization of such doping approach in practice.

## Competing interests

The authors declare that they have no competing interests.

## Authors' contributions

CC carried out the computation and wrote the manuscript. JHZ, GFD, HZS, and BYN provided technical assistance in computation. XJN, LZ, and JZ conceived and supervised the computation and discussed the results. CC and JZ co-wrote the manuscript. All authors read and approved the final manuscript.
